# No Evidence for a Role of Oral Contraceptive-Use in Emotion Recognition But Higher Negativity Bias in Early Follicular Women

**DOI:** 10.3389/fnbeh.2021.773961

**Published:** 2022-01-21

**Authors:** Ann-Christin Sophie Kimmig, Jasper Amadeus Bischofberger, Annika Dorothea Birrenbach, Bernhard Drotleff, Michael Lämmerhofer, Inger Sundström-Poromaa, Birgit Derntl

**Affiliations:** ^1^Department of Psychiatry and Psychotherapy, Tübingen Center for Mental Health (TüCMH), University of Tübingen, Tübingen, Germany; ^2^International Max Planck Research School for Cognitive and Systems Neuroscience, University of Tübingen, Tübingen, Germany; ^3^Institute of Pharmaceutical Sciences, University of Tübingen, Tübingen, Germany; ^4^Department of Women’s and Children’s Health, Uppsala University, Uppsala, Sweden; ^5^LEAD Graduate School and Research Network, University of Tübingen, Tübingen, Germany; ^6^Tübingen Neuro Campus, University of Tübingen, Tübingen, Germany

**Keywords:** sex hormones, facial emotion recognition, oral contraceptives, menstrual cycle, affective state

## Abstract

Accuracy in facial emotion recognition has shown to vary with ovarian hormones, both in naturally cycling women, as well as in women taking oral contraceptives. It remains uncertain however, if specific – endogenous and exogenous – hormonal levels selectively impact recognition of certain basic emotions (or neutral faces) and if this relationship coincides with certain affective states. Therefore, we investigated 86 women under different hormonal conditions and compared their performance in an emotion recognition task as well as self-reported measures of affective states. Based on self-reported cycle days and ovulation testing, the participants have been split into groups of naturally cycling women during their early follicular phase (fNC, *n* = 30), naturally cycling women during their peri-ovulatory phase (oNC, *n* = 26), and women taking oral contraceptives (OC, *n* = 30). Participants were matched for age and did not differ in education or neuropsychological abilities. Self-reported anxiety and depressive affective state scores were similar across groups, but current affective state turned out to be significantly more negative in fNC women. Independent of negative affective state, fNC women showed a significantly higher negativity bias in recognizing neutral faces, resulting in a lower recognition accuracy of neutral faces compared to oNC and OC women. In the OC group only, negative affective state was associated with lower recognition accuracy and longer response times for neutral faces. Furthermore, there was a significant, positive association between disgust recognition accuracy and negative affective state in the fNC group. Low progesterone levels during the early follicular phase were linked to higher negative affective state, whereas in the peri-ovulatory phase they were linked to elevated positive affective state. Overall, previous findings regarding impaired emotion recognition during OC-use were not confirmed. Synthetic hormones did not show a correlation with emotion recognition performance and affective state. Considering the important role of emotion recognition in social communication, the elevated negativity bias in neutral face recognition found for fNC women may adversely impact social interactions in this hormonal phase.

## Introduction

Women experience significant fluctuations of ovarian hormones over the menstrual cycle. Most notably, 17-β estradiol and progesterone levels change periodically (Becker et al., 2005). During the follicular phase at the beginning of the menstrual cycle estradiol and progesterone levels are low. Estradiol is rising until reaching its peak right before ovulation and abruptly decreasing with ovulation. During the luteal phase, progesterone is rising coinciding with a second yet smaller increase of estradiol, with both hormones declining during the late luteal phase reaching the initial low levels during menstruation. To prevent pregnancy and facilitate safe family planning, millions of women rely on hormonal contraceptives such as oral contraceptives (OCs) during their reproductive years (United Nations [UN], 2020). OCs typically contain ethinyl estradiol (synthetic estrogen) and progestin (synthetic progesterone) that effectively suppress endogenous estradiol and progesterone levels and thus ultimately prevent ovulation (Petitti, 2003). Evidence is accumulating that endogenous as well as synthetic ovarian hormones impact women’s socio-affective processing, including facial emotion recognition (Derntl et al., 2008a; Hamstra et al., 2014, 2015, 2017; for reviews see: Montoya and Bos, 2017; Lewis et al., 2019; Pahnke et al., 2019; Gamsakhurdashvili et al., 2021a).

For human communication, the perception and correct interpretation of facial expressions plays a major role. In a fast and direct way, facial expressions project the emotional state of a person to be perceived during social interactions (Horstmann, 2003). Among other functions, facial expressions of emotions can serve as eminent approach- or avoidance signals (Marsh et al., 2005). In naturally cycling (NC) women, several studies revealed superior facial emotion recognition in follicular compared to luteal NC women (Derntl et al., 2008a,b, 2013; Guapo et al., 2009; Rubin et al., 2011; for reviews see: Osório et al., 2018; Gamsakhurdashvili et al., 2021a). However, there are also some studies not finding any menstrual cycle effects on female’s emotion recognition (Rubinow et al., 2007; Zhang et al., 2013; Kamboj et al., 2015). These inconsistencies could potentially be explained by different levels of progesterone in the luteal NC women, as all studies that did not report a menstrual cycle effect measured women either during the early or late luteal phase in which progesterone levels are relatively lower than in the mid-luteal phase (Gamsakhurdashvili et al., 2021a). Within the follicular phase, first studies have not found a difference in emotion recognition skills between early follicular and late follicular (i.e., peri-ovulatory) NC women (Guapo et al., 2009; Zhang et al., 2013) except for fear recognition, for which women during the peri-ovulatory phase showed a better performance (Pearson and Lewis, 2005).

In some of these studies endogenous estradiol and progesterone levels were related to emotion recognition performance. Across cycle phases, estradiol was positively associated with facial recognition accuracy of fear (Pearson and Lewis, 2005) and sadness (Hamstra et al., 2017), whereas it was linked with lower performance in anger (Guapo et al., 2009) and disgust recognition (Kamboj et al., 2015). Contradictory findings were reported with respect to neutral face recognition, as it was positively linked to estradiol in one study (Hamstra et al., 2017), but negatively in another study (Shirazi et al., 2020). This incongruency could possibly be due to the inclusion of women in different cycle phases marked by different degrees of estradiol fluctuations as well as levels. In the early follicular phase, estradiol is comparatively low and stable, whereas in the peri-ovulatory phase levels are higher and rapidly fluctuating day by day. For progesterone, lower levels were linked to higher rates of misclassifying emotional faces as neutral (Derntl et al., 2008a; Kamboj et al., 2015). When including multiple cycle phases, progesterone was associated with an increased bias for negative emotions shown by higher recognition rates (Maner and Miller, 2014) as well as longer response times (Kamboj et al., 2015). However, progesterone levels have also been negatively linked with emotion recognition performance across cycle phases and specifically when only considering the luteal phase (Derntl et al., 2008a,^2013^). Therefore, the measurement timepoint in the luteal phase may indeed determine whether a higher sensitivity for negative emotions or a general lower face recognition rate can be detected compared to other cycle phases.

Like the midluteal phase, the hormonal milieu in OC-users is marked by a progestogen dominance as high doses of progestogens are needed to inhibit ovulation (Lovett et al., 2017). Therefore, it is not surprising that basic as well as complex facial emotion recognition was repeatedly found to be impaired in OC-users compared to NC women (Hamstra et al., 2014, 2015, 2017; Pahnke et al., 2019). These findings hold especially for negative emotions including anger, sadness, disgust (Hamstra et al., 2014, 2015, 2017). However, there are studies not reporting differences in emotion recognition performance between OC-users and NC women (Radke and Derntl, 2016; Gamsakhurdashvili et al., 2021b), including a large-scale study (*n* = 395; Shirazi et al., 2020). Interestingly, androgenicity of pill type seems to play no role in the impaired emotion recognition of OC-users (Pahnke et al., 2019). Regarding the modulatory role of endogenous and synthetic ovarian hormone levels not much is known, as previous studies have only measured endogenous but not exogenous ovarian hormone levels in blood or saliva samples. Since exogenous hormones pass the blood brain barrier and bind to hormone-receptors in brain regions involved with socio-emotional processing (Toffoletto et al., 2014; Barth et al., 2015; Louw-du Toit et al., 2017; Rehbein et al., 2021), including them in analyses could aid in shedding light on underlying mechanisms of facial emotion recognition during OC-use.

The aim of this study is to elucidate hormone-based differences in emotion recognition more closely by the incorporation of exogenous in addition to endogenous ovarian hormones. To assess the roles of estrogens and progestogens on facial emotion recognition largely independently, we included three groups of women with different hormonal states: (1) NC-women during the early follicular phase with low concentrations of estradiol and progesterone, (2) NC-women during the peri-ovulatory phase with high estradiol and low progesterone concentration, and (3) women actively taking combined OC-pills, with medium estrogen and high progestogen concentration. Based on previous literature on OC-and menstrual cycle-related differences, we hypothesize that: (1) OC-users show impaired emotion recognition relative to NC women (see for reviews: Osório et al., 2018; Gamsakhurdashvili et al., 2021a), and for NC women, we hypothesized that: (2) Women in the peri-ovulatory phase show enhanced fear recognition compared to early follicular NC women (Pearson and Lewis, 2005), whilst there is no evidence for an altered fear recognition in OC compared to NC women.

We aim for a systematic investigation of hormone-related effects on female’s facial recognition performance. Therefore, we ran explorative analyses with regards to – especially synthetic – ovarian hormones. In addition, affective state supposedly impacts the recognition of valence-congruent emotions but impairs performance for valence-incongruent facial expressions (Schmid and Schmid Mast, 2010). Moreover, current affective state has been associated with fluctuations of ovarian hormones (Reed et al., 2008; Ocampo Rebollar et al., 2017). To account for a possible interplay of affective state and ovarian hormones on emotion recognition performance, we not only exploratively checked for relations of affective state with emotion recognition performance in different hormonal states, but also to ovarian hormone levels.

## Materials and Methods

To investigate hormone-related differences in facial emotion recognition, we used a quasi-experimental, cross-sectional study design.

### Participants

A total of 86 healthy females aged between 18 and 33 years (m_age_ = 23.8, ± 3.1) were recruited via postings at the University of Tübingen, the University Hospital Tübingen, social media, as well as in gynecological practices in Tübingen. Based on self-reported cycle days, the women were divided into three groups: (1) women with long-term (>6 months) OC-use (OC group; *n* = 30, m_age_ = 23.6 ± 3.0), (2) NC-women (>4 months) during the early follicular phase (fNC group; *n* = 30, m_age_ = 23.8 ± 3.3), and (3) NC-women (> 4 months) during the peri-ovulatory phase (oNC group; *n* = 26, m_age_ = 24.0 ± 3.0). The assignment to hormonal status groups was validated by female sex-hormone measurement and described in the Results section (“Sample Description and Hormonal Levels”). The sample size (*n* = 86) was based on previous, conceptually related studies (Derntl et al., 2013; Radke and Derntl, 2016; Dan et al., 2019; Gurvich et al., 2020; Kimmig et al., 2021). The fNC group was tested 2–5 days after the onset of their menses, the oNC group 3 days before until 2 days after a positive increase of the luteinizing hormone confirmed via LH test (nal van minden GmbH, Germany). The OC group was tested during day 3–21 of active pill intake, expecting to have steady, suppressed estradiol- and progesterone levels. None of the participants were diagnosed with a gynecological illness nor had a lifetime pregnancy. All women gave informed consent, and the study was approved by the ethics committee of the Medical Faculty of the University of Tübingen (331/2016BO2).

An overview of sociodemographic and neuropsychological characteristics and the plasma hormone profiles for the different groups is provided in Table 1.

**TABLE 1:** Sample characteristics (mean and standard deviation if not otherwise specified) and hormone profiles per group (median and interquartile range).

	OC	fNC	oNC	*p*-value
*N*	30	30	26	
Age (years)	23.6 (3.0)	23.8 (3.3)	24.0 (3.0)	0.906
Education (l/m/h)^1^	1/20/9	0/20/10	1/15/10	0.854
Verbal intelligence (WST, raw scores)	32.4 (2.4)	32.9 (3.1)	32.7 (2.4)	0.563
Cognitive flexibility (TMTB-A, sec)	18.2 (9.9)	16.8 (9.7)	16.4 (7.8)	0.718
Depressive mood (BDI-II, scores)	5.5 (4.3)	7.4 (4.1)	5.2 (3.5)	0.072
Social anxiety (Mini-Spin-R)	7.5 (2.9)	7.9 (1.6)	7.2 (2.2)	0.247
Trait anxiety (STAI)	34.1 (8.6)	34.5 (6.9)	32.8 (6.8)	0.648
State anxiety (STAI)	33.8 (7.1)	35.7 (7.0)	33.8 (8.7)	0.521
Positive affective state (PANAS)	21.3 (8.3)	23.7 (5.9)	24.1 (5.5)	0.308
Negative affective state (PANAS)	2.9 (3.8)	5.3 (4.7)	2.7 (3.0)	0.026 fNC > OC
**Hormone profiles**				
EndoE2 (pmol/L)	16.9 (7.0)	98.4 (45.2)	444.2 (462.2)	<0.001 oNC > fNC > OC
ExoE2 (pmol/L)	72.7 (36.3)			<0.001^2^ oNC > fNC, OC
EndoP (nmol/L)	0.1 (0.6)	0.3 (0.4)	1.0 (4.4)	<0.001 oNC > fNC > OC
ExoP (nmol/L)	33.6 (37.2)			<0.001^3^ OC > oNC > fNC
Testosterone (nmol/L)	0.7 (0.4)	0.7 (0.4)	0.9 (0.5)	<0.001 oNC > OC, fNC

*^1^l, no higher education entrance qualification; m, higher education entrance qualification; h, university degree.*

*^2,3^Group differences between endogenous hormone levels for NC women and exogenous hormone levels of OC-users calculated. WST – Wortschatztest; TMT – Trial-making test; BDI – Beck’s depression inventory; SPIN-R – social phobia inventory revised; STAI – state-trait anxiety inventory; PANAS – positive and negative affect schedule; EndoE – endogenous estradiol; exoE – exogenous estradiol; endoP – endogenous progesterone; exoP – exogenous progesterone.*

### Procedure

Participants came in for two appointments: (1) a screening (45–60 min) and (2) an experimental session (30–45 min). After a mental health screening, all women performed neuropsychological tests and reported sociodemographic information during the first session. The experimental session took place in the respective hormonal phase (i.e., active OC intake, early follicular or peri-ovulatory phase). At its beginning, participants rated their current affective state. Subsequently, the emotion recognition task was performed. After task completion, plasma samples (2 × 9 ml EDTA) were taken by trained medical staff to obtain the actual hormone status. At the end of the session, participants filled in several questionnaires including state-trait anxiety and depressive mood.

### Materials and Measures

#### Emotion Recognition Task

Stimuli consisted of 36 colored pictures of European-American faces showing five basic emotions (happiness, sadness, anger, fear, and disgust) as well as neutral expressions (i.e., six items per condition, see Gur et al., 2002 for stimulus material). This is a short version of the Vienna Emotion Recognition Task (VERT-K) which has already successfully been carried out to investigate female emotion recognition under varying ovarian hormone concentrations (Derntl et al., 2008a,b, 2013; Radke and Derntl, 2016). In each trial, participants were instructed to choose the correct emotion from six verbal possibilities presented in a random order next to the target face stimulus by button press. A response was necessary to finish the trial. The sequence of stimuli presentation was pseudo-randomized for emotion type and sex of actor. Intertrial intervals lasted 1 s. The variables of interest were emotion recognition accuracy and response time. In total, the task lasted about 2–4 min.

#### Neuropsychological Tests and Questionnaires

Positive and negative affective state was assessed using the Positive and Negative Affect Scale (PANAS; Watson et al., 1988). Current affective state was included to control for potential confounding effects on emotion recognition. Moreover, we were interested in the interplay of affective state, hormone status and emotion recognition.

The following measures were used for sample characterization and assessing comparability of the hormonal status groups. The absence of current or lifetime mental disorders was checked using a semi-structured interview (SCID screening; Wittchen et al., 1997). The Wortschatztest (WST; Schmidt and Metzler, 1992) was used to assess verbal intelligence and the Trail-Making-Test A and B (TMT; Reitan, 1992) measured cognitive flexibility. Furthermore, several affective measures were taken including state-trait anxiety (STAI-I; Laux et al., 1981), social anxiety with the brief version of the Social Phobia Inventory (Mini-SPIN-R; Aderka et al., 2013) and depressive mood using the Beck’s depression inventory (BDI; Hautzinger et al., 2006). These neuropsychological and psychopathological measures were used for sample characterization and assessing comparability of the hormonal status groups.

#### Hormone Assessment

After blood collection, the sample was centrifuged to obtain plasma, which was aliquoted into microtubes and stored at –70°C. Liquid chromatography-tandem mass spectrometry (LC-MS/MS) was used to determine hormone levels of estradiol (endoE), progesterone (endoP), testosterone, and ethinylestradiol (exoE) as well as progestins (exoP) in pg/mL. Plasma concentrations of the progestins were determined individually for dienogest, levonorgestrel, nomegestrol as well as chlormadinone acetate. The analytical system consisted of a 1290 Infinity II UHPLC (Agilent Technologies, Germany) coupled to a QTRAP 4500 mass spectrometer (Sciex, United States). The hormones were quantified via a surrogate calibrant approach (Li and Cohen, 2003; Drotleff et al., 2018) and the method was validated according to FDA guidelines. The dynamic range of endoE, endoP, testosterone, exoE and the various progestins ranged from 3.45–5179.13, 1.0–47657, 1.9–11438.00, 2.0–3000, and 10–20000 pg/mL, respectively. To evaluate the performance of the method and document the validity of the analytical measurements method, quality control samples (QCs) were analyzed on three consecutive days. Interday precision (i.e., repeatability between different days) and accuracy (as % recovery of QCs’ nominal concentration) were 7.0–9.1% and 96.8–100.5% (endoE), 6.4–9.9 and 97.0–104.6% (endoP), 7.4–9.9% and 94.3–106.5% (testosterone), 5.6–12.3% and 97.1–99.9% (exoE), as well as 4.4–11.1% and 93.4–109.2% (progestins), indicating excellent method performance within the acceptance criteria of the FDA bioanalytical method validation guideline. Interday precision measures the repeatability of the concentrations of the quality control samples between different days and interday accuracy the percent recovery (% found/nominal concentration) in the quality control samples on the different days.

### Statistical Analyses

All statistical analyses were performed using SPSS 25 (IBM SPSS Statistics) with alpha set to 0.05, if not otherwise specified. All *post hoc* analyses were Bonferroni corrected.

#### Sample Characteristics and Hormonal Levels

Group differences (OC, fNC, and oNC) in sociodemographic (i.e., age and educational level), neurocognitive (i.e., verbal intelligence and cognitive flexibility) and affective parameters [i.e., affective states (PANAS), state and trait (social) anxiety (STAI and mini-SPIN), as well as depressive mood (BDI)] were either analyzed with an independent ANOVA (age and state anxiety, normality: yes, homogeneity of variances: yes), a Welch’s ANOVA (positive affective state, normality: yes, homogeneity of variances: no) or a non-parametric Kruskal-Wallis test [verbal intelligence, cognitive flexibility, negative affective state, trait (social) anxiety and depressive mood, normality: no, homogeneity of variances: yes]. Educational level is a categorical variable (i.e., 1 – no higher education entrance qualification, 2 – higher education entrance qualification, 3 – university degree) and thus analyzed with Fisher’s exact test, as not all cells had counts higher than 5.

For OC-users, only exogenous hormone levels were used for analyses as endogenous hormones are suppressed to very low levels. All hormones (endogenous levels for NC groups and exogenous levels of P for OC-users), except for testosterone and exoE, were analyzed using the non-parametric median test, as normality (according to visual inspection and Kolmogorov Smirnov test: *p* < 0.05) as well as homogeneity of variances (Levene’s test: *p* < 0.05) were not given. Group differences of testosterone and exoE were assessed with the Kruskal-Wallis test (normality: no, homogeneity of variances: yes).

#### Emotion Recognition Accuracy

The number of correct responses was calculated for each target emotion, resulting in a mean score of emotion recognition accuracy (percent correct) for each participant per emotion. Kolmogorov-Smirnov tests suggested that the data was not normally distributed (*p* < 0.05). We therefore used Generalized Estimating Equations (GEE) with emotion as within-subject factor (anger, fear, happiness, sadness, disgust, and neutral) and hormonal group as between-subjects factor (fNC, oNC, and OC) to analyze differences in emotion recognition performance dependent on hormonal status. Significant effects were followed up with Bonferroni-corrected pairwise comparisons. As the groups showed significantly different baseline levels in the scores of the negative affective state scale (PANAS, see section “Sample Description and Hormonal Levels”), we additionally performed an ANCOVA with emotion as within-subject factor, group as between-subjects factor and negative affective states scores as covariate.

#### Emotion Recognition Response Times

Like the accuracy measure, mean emotion recognition response times were also calculated per emotion for each participant. However, only correct trials and trials with response times larger than 200 ms were considered. A mixed AN(C)OVA with emotion as within-subject factor, group as between-subjects factor and negative affective state as covariate was performed. Due to the violation of the sphericity assumption (Maulchy’s test: *p* = 0.045), Huynh-Feldt corrected statistics were reported (Greenhouse ε > 0.75). Bonferroni corrected pairwise comparisons were used as *post hoc* analyses.

#### Correlational Analyses

Within group associations between overall emotion recognition accuracy and response times with self-reported affective state (i.e., PANAS positive and negative scales) and hormones (endogenous for NC groups, exogenous for OC-users; concentrations of ovarian sex hormones as well as testosterone) were investigated. Besides correlation analyses using the total percent correct for emotion recognition accuracy and total mean response time, exploratory analyses for single emotions were carried out if the GEE or ANOVA analyses revealed significant emotion-specific group differences. Normally distributed data was analyzed with Pearson correlations (OC: overall and neutral emotion recognition response times, exoE, and positive affective state; fNC: overall emotion recognition response time, testosterone, endoE and positive affective state; oNC: overall response time, testosterone, endoE, and positive affective state), whereas Spearman Rank correlations (*rho*_*s*_) were used to account for non-normality in all other correlational analyses.

## Results

### Sample Description and Hormonal Levels

Women across the different hormonal status groups did not differ on sociodemographic characteristics such as age [*F*(2,83) = 0.10, *p* = 0.906] and educational level [*p* = 0.854 (Fisher’s exact test)]. Furthermore, the groups were similar for neuropsychological parameters including verbal intelligence, cognitive flexibility, depressive mood as well as (social) anxiety (all |*H*| ≤ 5.26, all *p* ≥ 0.072). Baseline levels of state anxiety and positive affective state at the beginning of the experimental session were comparable amongst women in different hormonal phases (all |*F*| ≤ 1.20, all *p* ≥ 0.308), whereas fNC women reported significantly higher negative affective state compared to OC-users [main effect: *H*(2) = 7.28, *p* = 0.026; fNC > OC: *p* = 0.044; fNC > oNC: *p* = 0.088; OC > oNC: *p* = 1.00].

**FIGURE 1:**
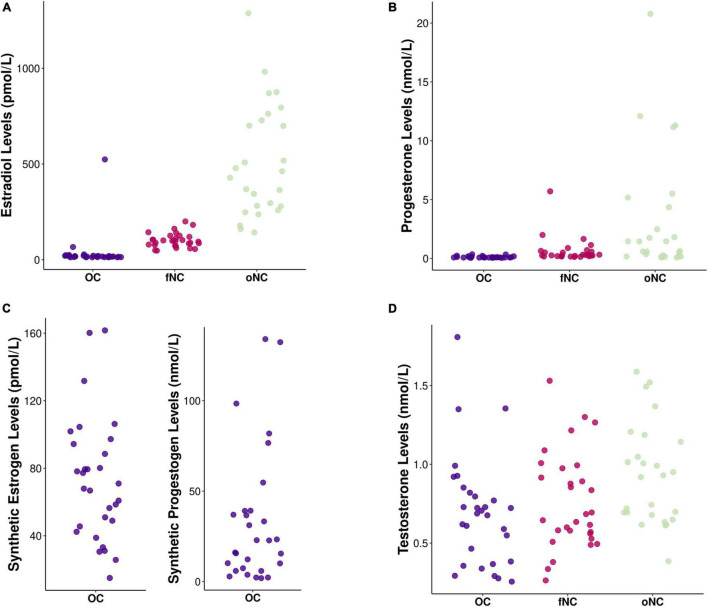
Chart depicting hormone levels of **(A)** endogenous estradiol (in pmol/L), **(B)** endogenous progesterone (in nmol/L), **(C)** exogenous (i.e., synthetic) estrogens (in pmol/L) and progestogens (in nmol/L), and **(D)** endogenous testosterone (in nmol/L) for each hormonal status group [i.e., OC – oral contraceptive users (blue), fNC – naturally cycling women in early follicular phase (magenta), and oNC – naturally cycling women in periovulatory phase (light green)].

Figure 1 depicts the hormonal levels of the different hormonal status groups. Hormonal analyses using median tests confirmed that the women assigned to the respective groups indeed differed in hormonal profiles accounting for endogenous as well as for exogenous sex hormones [EndoE2 vs. ExoE2: *H*(2) = 56.73, *p* < 0.001; EndoP vs. ExoP: X^2^(2) = 49.58, *p* < 0.001; Testosterone: *H*(2) = 7.92, *p* = 0.019]. As expected, the oNC group had significantly higher levels of estrogens than the OC and fNC group (both *p* < 0.001, OC vs. fNC: *p* = 0.097), whereas the OC group had highest levels of progestogens (OC > oNC, fNC: both *p* < 0.001), followed by the oNC group (oNC > fNC: *p* = 0.022). Testosterone was significantly lower in OC-users compared to the oNC group (*p* = 0.020).

### Emotion Recognition Accuracy

The GEE analysis for the emotion recognition accuracy (i.e., percent correct) revealed a main effect of emotion [Wald-X^2^(5) = 468.52, *p* < 0.001, see Table 2 for means]. After Bonferroni correction, recognition rates of all emotions differed significantly from each other (all *p* ≤ 0.026) except for happiness vs. anger (*p* = 1.000), anger vs. fear (*p* = 0.273), and fear vs. neutral (*p* = 1.000). Happy and angry faces were recognized best, whilst disgusted and sad expressions had the lowest performance scores.

**TABLE 2:** Emotion recognition performance (in percent) and response times (in ms) across the whole sample and for the individual hormonal groups (presented as mean and standard deviation).

	Whole sample (*n* = 86)	OC (*n* = 30)	fNC (*n* = 30)	oNC (*n* = 26)
**Emotion recognition response accuracy (%)**				
Happiness	96.1 (9.1)	96.7 (8.1)	96.1 (11.3)	95.5 (7.5)
Anger	95.4 (11.3)	95.6 (8.7)	97.2 (6.3)	93.6 (11.6)
Fear	91.1 (12.2)	91.1 (12.2)	88.3 (13.9)	94.2 (9.4)
Disgust	76.2 (17.6)	78.3 (15.3)	77.2 (18.8)	72.4 (18.8)
Sadness	61.6 (23.2)	61.1 (24.1)	63.3 (18.3)	60.3 (27.5)
Neutral	89.7 (15.4)	93.9 (11.1)	82.2 (19.0)	93.6 (11.6)
**Emotion recognition response times (ms)**				
Happiness	2204.2 (629.0)	2104.6 (757.4)	2212.7 (428.1)	2309.3 (658.6)
Anger	2837.7 (937.8)	2579.0 (744.9)	2943.6 (966.6)	3013.9 (1066.1)
Fear	3510.4 (1265.2)	3308.5 (977.2)	3628.3 (1459.6)	3607.4 (1337.0)
Disgust	3008.8 (1095.7)*	2868.2 (1185.2)	3200.0 (1118.2)	2953.9 (958.3)*
Sadness	3135.1 (952.1)*	3177.5 (783.6)	3225.5 (1159.3)*	2987.0 (884.3)
Neutral	2570.9 (830.5)*	2327.0 (543.5)	2596.2 (867.1)*	2824.0 (997.0)

*One participant missing as no correct answers were recorded.

**FIGURE 2:**
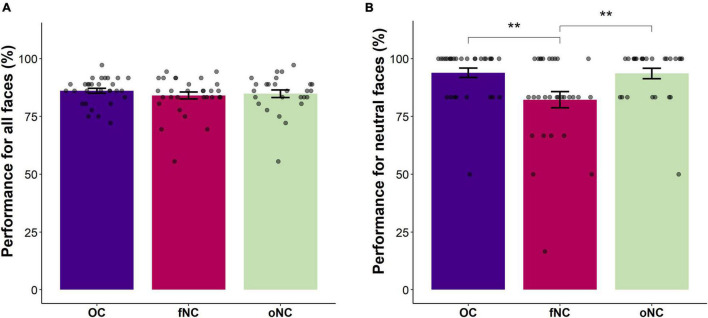
Bar chart depicting the **(A)** overall emotion recognition accuracy (in percent) and **(B)** the emotion recognition accuracy for neutral faces (in percent) per group [i.e., OC – oral contraceptive users (blue), fNC – naturally cycling women in early follicular phase (magenta), and oNC – naturally cycling women in periovulatory phase (light green)]. Error bars with 1 SE. **p < 0.01.

Contrary to our expectation, there was no main effect of group [Wald-X^2^(2) = 1.39, *p* = 0.500, see Figure 2A]. However, the interaction emotion-by-group turned out significant [Wald-X^2^(10) = 25.34, *p* = 0.005]. To disentangle the significant interaction, separate GEEs for each group were performed. For the OC and the oNC women, recognition performance of disgust and sadness were significantly worse than for the remaining emotions (all *p* ≤ 0.001). Whereas OC women recognized sadness significantly worse than disgust (*p* = 0.001), oNC women’s recognition accuracy did not differ between sad and disgust expressions (*p* = 0.095). Happy, angry, neutral, and fearful faces were equally well recognized (all *p* ≥ 0.116, except for happy vs. fear in OC: *p* = 0.014). In contrast, fNC women recognized facial expressions of anger and happiness significantly better than fearful, neutral, disgusted, and sad faces (all *p* ≤ 0.006). Sad expressions showed the lowest accuracy (all *p* ≤ 0.006) in fNC women. Fear was significantly better recognized than disgust (*p* = 0.002), accuracy for neutral faces did not differ significantly from either of the two emotions (all *p* ≥ 0.116). Overall, the recognition order per emotion for OC and oNC women was happy, angry, neutral, (>) fearful > disgust, (>) sad. Whereas fNC women’s recognition order was angry, happy > fear, neutral (not different from fear or disgust), > disgust > sad. Therefore, the recognition of neutral faces presents the largest difference in the order of emotion recognition between the groups. Congruently, separate GEE analyses looking at between group difference for the specific emotions, revealed no group difference for the five basic emotions (all |Wald-X^2^| ≤ 3.84, *p* ≥ 0.147), while for neutral faces a significant group difference emerged [Wald-X^2^(2) = 9.57, *p* = 0.008]. The fNC women had significantly lower accuracy rates for the neutral condition than OC and oNC women (all *p* ≤ 0.005, see Figure 2B). Neutral faces were mostly misclassified by fNC women as sad or angry instead (66 and 25% of incorrect trials, respectively).

When directly testing for our directed second hypotheses in a GEE only involving fear and the two NC groups, we indeed observed superior fear recognition in oNC compared to fNC women [Wald-X^2^(1) = 3.66, *p*_1*tailed*_ = 0.028].

Adding negative affective state as a covariate did not change the aforementioned results (except that disgust = sadness for fNC: *p* = 0.072) and had no direct link with emotion recognition accuracy [negative affective state main effect: Wald-X^2^(1) = 0.01, *p* = 0.924; negative affective state-by-emotion: Wald-X^2^(5) = 3.13, *p* = 0.680]. However, there was a significant group-by-affective state-by-emotion interaction [Wald-X^2^(10) = 21.34, *p* = 0.019]. Separate emotion-specific GEE analyses revealed no interaction effect with negative affective state for recognition of all emotional faces as well as for neutral faces (all |Wald-X^2^| ≤ 2.57, *p* ≥ 0.277), except for disgust [group-by-affective state: Wald-X^2^(2) = 6.03, *p* = 0.049]. Parameter estimates suggest that negative affective state had a significantly larger positive effect on disgust recognition in fNC compared to oNC women [Wald-X^2^(1) = 5.98, *p* = 0.014], whereas OC-users did not differ from either NC group (all |Wald-X^2^| ≤ 3.05, *p* ≥ 0.081). Negative affective state was positively related with accuracy in the fNC group [*rho*_*s*_(30) = 0.38, *p* = 0.036], whereas there were no significant correlations for the OC [*rho*_*s*_(30) = −0.10, *p* = 0.617] and the oNC group [*rho*_*s*_(26) = −0.30, *p* = 0.129].

### Emotion Recognition Response Times

The mixed ANOVA design of emotion recognition response times only from correct trials revealed a significant main effect of emotion [*F*(4.9,385.0) = 23.97, *p* < 0.001]. *Post hoc* analyses revealed that happy faces were recognized the fastest (all *p* ≤ 0.027), followed by neutral faces (all *p* ≤ 0.027, but neutral vs. angry: *p* = 0.074). Angry and disgusted expressions were significantly faster recognized than fearful (*p* = 0.001 and *p* = 0.043, respectively), but not sad faces (all *p* ≥ 0.208). There was no main effect of group [*F*(2,79) = 0.93, *p* = 0.401] nor a group-by-emotion interaction [*F*(9.8,385.0) = 0.73, *p* = 0.695] in the response times including only correct trials.

Adding negative affective state as a covariate did not affect the findings reported above and also had no significant relation with emotion recognition in women with different hormonal states [negative affective state main effect: *F*(1,77) = 0.90, *p* = 0.346; negative affective state-by-emotion interaction: *F*(4.9,376.5) = 1.46, *p* = 0.203].

### Within-Group Correlational Analyses: Emotion Recognition, Sex Hormones, and Self-Reported Affective Measures

Correlational analyses were run to assess whether affective states (positive and negative) or hormone levels (endogenous and exogenous ovarian hormones for NC women and OC-users, respectively) are related to emotion recognition performance (i.e., accuracy and response times) within different hormonal states. All correlations between overall accuracy and response times with sex hormones and self-reported positive and negative affective state remained non-significant (all |*r*_(s_*_)_*| ≤ 0.36, all *p* ≥ 0.073). Since there was a significant difference in emotion recognition accuracy of neutral faces between OC and fNC women, within group correlations between sex hormones, self-reported affective state measures and emotion recognition parameters of neutral faces have been additionally computed. In OC-users, lower negative affective state was associated with higher recognition accuracy [*rho*_*s*_(29) = −0.49, *p* = 0.008] and faster response times [*rho*_*s*_(29) = 0.41, *p* = 0.028], when presented with neutral faces. None of the other self-report measures and sex hormone levels correlated significantly with emotion recognition of neutral faces in the OC group (all |*r*_(s_*_)_*| ≤ 0.36, all *p* ≥ 0.054). There were no significant correlations for the fNC group regarding the recognition of neutral faces (all |*rho*_s_| ≤ 0.27, all *p* ≥ 0.143). Fear recognition accuracy was not significantly related to affective states or hormonal levels in the fNC and oNC groups (all |*rho*_s_| ≤ 0.32, all *p* ≥ 0.087). Fear recognition response times showed a positive association with testosterone levels in fNC women [*rho*_*s*_(30) = 0.46, *p* = 0.012], whilst all other correlation remained non-significant in both NC groups (all |*rho*_s_| ≤ 0.30, all *p* ≥ 0.143).

Furthermore, we were interested whether hormone-levels (endogenous and exogenous ovarian hormones in NC women and OC-users, respectively) were related to positive or negative affective state, which in turn could be related to emotion recognition. Spearman rank correlations revealed a negative association of progesterone with negative affective state [*rho*_*s*_(30) = −0.47, *p* = 0.009] in the fNC group, whereas in the oNC group endoP correlated negatively with positive affective state [*rho*_*s*_(26) = −0.63, *p* = 0.001]. Outlier removal did not alter results significantly. All remaining correlations between sex hormones and affective state measures did not reach significance (all |*r*_(s_*_)_*| ≤ 0.26, all *p* ≥ 0.209).

## Discussion

Emotion recognition and other socio-emotional processes have been repeatedly suggested to be associated with fluctuations of endogenous sex hormones as well as with the intake of synthetic ovarian hormones (see reviews: Montoya and Bos, 2017; Osório et al., 2018; Lewis et al., 2019; Gamsakhurdashvili et al., 2021a). However, studies are not entirely conclusive, and the underlying mechanisms remain largely unclear. Therefore, our aim was to systematically investigate the role of hormonal status in facial emotion recognition by linking performance not only to endogenous hormones in NC women, but for the first time also to the more dominant exogenous hormone levels in OC-users. Here the use of the highly recommended LC-MS method for hormone determination is a major strength of this study. Furthermore, we investigated associations to other emotional processes which could impact emotion recognition such as negative and positive affective state.

Overall, women during the early follicular phase, independently of negative affective state differences among groups, showed specific deficits in recognizing neutral faces by misjudging neutral faces as sadness or anger. Furthermore, in a direct comparison peri-ovulatory women, as expected, recognized fearful faces significantly better than early follicular women. There were no significant group-related differences in emotion recognition response times. Endogenous and exogenous sex hormones were, not linked to overall or neutral recognition performance. During the early follicular phase low progesterone levels were linked to higher negative affective state. Notably, during the peri-ovulatory phase progesterone levels were negatively associated with positive affective state.

Contrary to our expectation and previous literature (Hamstra et al., 2014, 2015, 2017; Pahnke et al., 2019), we were not able to replicate inferior emotional face recognition performance in OC-users compared to NC women. Even though studies use the same tasks for basic (i.e., VERT as in the present study) or complex (i.e., Reading the mind in the eye task) emotion recognition, they reveal mixed results. In line with our findings, Radke and Derntl (2016) found no OC-related impairment in basic emotion recognition. Furthermore, the up-to-now largest study on hormonal contraceptives and complex emotion recognition also failed to find any significant differences (Shirazi et al., 2020). This incongruency in findings could be due to the interplay of OC-use with other potential modulatory factors. For instance, Hamstra et al. (2016) found only a significant impairment in emotion recognition relative to NC women in OC-users with a certain genotype of mineralocorticoid receptor (MR-haplotype 1/3). In the present study, we found negative affective state to play a role in neutral face recognition of OC-users. The worse their affective state was, the more likely women misclassified a neutral expression as sadness or anger (i.e., increased negativity bias). The lack of finding any significant associations between endogenous and exogenous hormone levels with emotion recognition performance in OC-users further corroborates the view that OC-related effects may be complex and mediated rather than straightforward.

Regarding menstrual cycle phases, in a direct comparison, we replicated previous findings indicating superior fear recognition in the peri-ovulatory phase compared to the early follicular phase with significantly lower endoE2 levels (Pearson and Lewis, 2005). Interestingly, this superior fear recognition does not translate into increased fear processing in peri-ovulatory women. In fact, high levels of endoE2 have been linked to enhanced fear extinction, whereas low levels (i.e., in the early follicular phase) were associated with enhanced fear conditioning (Montoya and Bos, 2017). Moreover, high levels of estradiol were previously associated with lower disgust (Kamboj et al., 2015) and anger recognition (Guapo et al., 2009), accordingly peri-ovulatory women had lower accuracy in recognizing these facial expressions than early follicular women, however, these differences were only descriptive and did not reach significance. In the early follicular group, disgust recognition accuracy was positively associated with negative affective state. This is congruent with the notion that affective state may enhance emotion recognition of valence-congruent emotions (Schmid and Schmid Mast, 2010). In OC-users and peri-ovulatory women the negative affective state may have not been pronounced and variable enough to reveal such associations.

Independent of negative affective state, early follicular women were significantly worse than OC-users and peri-ovulatory NC women in recognizing neutral faces. The neutral faces were mostly misclassified as being sad or angry instead. However, there were no significant differences in response times. Therefore, suggesting that fNC women were not aware of their difficulty in recognizing these faces correctly, as if they were uncertain, response times should be longer. In previous studies, low endoP levels were associated with a higher number of stimuli falsely classified as neutral (Derntl et al., 2008a), faster response times in correctly identifying neutral faces (Kamboj et al., 2015), and higher amygdala activation during neutral face processing (Derntl et al., 2008b). Therefore, from these studies we could have expected enhanced neutral face processing of early follicular women, as here endogenous progesterone is low. However, instead we found a greater negativity bias (i.e., misjudging neutral as negative expressions) in this group, which was however not related to ovarian hormone concentrations. This incongruency could be explained by the different menstrual cycle phases included in the studies. The previous studies (Derntl et al., 2008a,b; Kamboj et al., 2015) pooled follicular and luteal women to generate hormone correlations. Therefore, these findings could be rather driven by the inclusion of luteal women with high progesterone levels. Furthermore, the early follicular phase was largely underrepresented in the follicular groups of the previous samples, making a comparison of the previous studies with the present study difficult. Negativity biases in neutral or ambiguous face recognition have been repeatedly implicated in individuals with affective disorders, (social) anxiety and other mental problems (Richards et al., 2002; Leppänen et al., 2004; Yoon and Zinbarg, 2008; Mier et al., 2014; Münkler et al., 2015; Gutiérrez-García and Calvo, 2017; Peschard and Philippot, 2017). These biases or overinterpretations could contribute to the difficulties in social interactions and relations in these individuals. Our analyses, however, revealed no link between affective state and (social) anxiety measures with the negativity bias in the early follicular group. Considering, that the fNC women had no elevated or clinically relevant levels on these scales, these null finding may not be surprising. Nevertheless, fNC women could have felt more menstrual discomfort and pain, which is not evaluated by the PANAS or the STAI, causing a greater precaution in processing of neutral facial expression to account for their increased vulnerability. Indeed, there is evidence of negative interpretation biases associated with pain (Khatibi et al., 2015; Heathcote et al., 2016). Therefore, future studies are needed to examine more closely the possible link between menstrual pain/discomfort and negative interpretation biases.

Independent of emotion recognition, our findings support previous literature reporting a link between menstrual cycle and affective state (Reed et al., 2008; Ocampo Rebollar et al., 2017). As similarly shown by Ocampo Rebollar et al. (2017), the negative link between progesterone and positive affective state in the peri-ovulatory phase implies that pre-ovulatory women have more positive affective state which decreases as ovulation comes closer and progesterone levels start rising. In the early follicular phase, however, lower levels of progesterone were linked with worse affective state. Since progesterone levels in this phase are already low, even lower concentrations could lead to an interruption of the mood stabilizing effects of its metabolite allopregnanolone, by reducing its effect (through lower concentrations) on the GABAeric system (Chen et al., 2021). However, these findings ought to be interpreted with caution, given the generally low levels of progesterone in the follicular phase.

In this study we have only included women using OCs but excluded women using other (hormonal) contraception methods such as intrauterine devices or vaginal ring. To fully capture the impact of (hormonal) contraception on emotion recognition and more general socio-emotional abilities, future studies should systematically investigate their effect. Furthermore, our study investigated women’s emotion recognition in a cross-sectional design, comparing different women with different hormonal status once. However, a longitudinal design enabling a within-subject comparison would be beneficial to better characterize the impact of endogenous and exogenous hormones on behavioral outcomes. Additionally, statistical power could be improved this way, without really having to increase the sample size (Gonzales and Ferrer, 2016). Another downside of the sample size per hormonal status group in addition to non-linearity issues of the data was that no mediation analyses using affective state could be carried out to investigate the interplay of sex hormones and affective state on emotion recognition (minimum size per group *n* = 72 for medium effects; Fritz and Mackinnon, 2007). Finally, in this study we only measured emotion recognition of basic emotions. Since in real-life, emotion recognition of complex next to basic emotions plays a major role, the inclusion of complex emotions in the study design could have raised ecological validity.

## Conclusion

With the current study we shed some light on the role of different hormonal conditions (i.e., OC-use, early follicular and peri-ovulatory phase) in emotion recognition abilities of women. Our results suggest that women in their early follicular phase show both, elevated negative affective state as well as a negativity bias in perceiving neutral faces (i.e., neutral misjudged as sadness or anger), which may impair their success in social interactions. Furthermore, in a direct comparison peri-ovulatory women showed better fear recognition accuracy. Generally, we were not able to replicate OC-related impairments in emotion recognition performance. More importantly, we also found no significant links between endogenous and exogenous hormone levels with emotion recognition, suggesting a more complex mechanism by which emotion recognition is possibly influenced by hormonal contraception. Thus, the study motivates more research to better understand how different hormonal conditions do impact women’s social life, and ultimately their mental health. A better understanding of these processes is necessary to provide gynecologists and potential users with details on potential consequences of hormonal contraceptives on female social cognition.

## Data Availability Statement

The raw data supporting the conclusions of this article will be made available by the authors, without undue reservation.

## Ethics Statement

The studies involving human participants were reviewed and approved by Ethics Committee of the Medical Faculty of the University Tübingen. The patients/participants provided their written informed consent to participate in this study.

## Author Contributions

A-CSK and BiD designed the study and supervised data collection. BiD and IS-P helped with the methodological setup. A-CSK and ADB collected data. BiD, IS-P, and JAB were involved in the planning of data analysis and interpretation of data. BeD carried out all hormone detection analyses under the supervision of ML. A-CSK performed data analyses and wrote the manuscript. All authors contributed to the manuscript.

## Conflict of Interest

The authors declare that the research was conducted in the absence of any commercial or financial relationships that could be construed as a potential conflict of interest.

## Publisher’s Note

All claims expressed in this article are solely those of the authors and do not necessarily represent those of their affiliated organizations, or those of the publisher, the editors and the reviewers. Any product that may be evaluated in this article, or claim that may be made by its manufacturer, is not guaranteed or endorsed by the publisher.
